# Blockade of prostaglandin E2 receptor 4 ameliorates peritoneal dialysis-associated peritoneal fibrosis

**DOI:** 10.3389/fphar.2022.1004619

**Published:** 2022-11-11

**Authors:** Qimei Luo, Mi Liu, Yanhong Tan, Jinzhong Chen, Wei Zhang, Shaoxin Zhong, Jianyi Pan, Qingkun Zheng, Lewei Gong, Lijuan Su, Zhanjun Jia, Xianrui Dou

**Affiliations:** ^1^ Department of Nephrology, Shunde Hospital, Southern Medical University (The First People’s Hospital of Shunde), Foshan, China; ^2^ Nanjing Key Laboratory of Pediatrics, Children’s Hospital of Nanjing Medical University, Nanjing, China

**Keywords:** prostaglandin E2 receptor 4, ONO-AE3-208, peritoneal dialysis, fibrosis, inflammation

## Abstract

Inflammatory responses in the peritoneum contribute to peritoneal dialysis (PD)-associated peritoneal fibrosis. Results of our previous study showed that increased microsomal prostaglandin E synthase-1-mediated production of prostaglandin E2 (PGE2) contributed to peritoneal fibrosis. However, the role of its downstream receptor in the progression of peritoneal fibrosis has not been established. Here, we examined the role of PGE2 receptor 4 (EP4) in the development of peritoneal fibrosis. EP4 was significantly upregulated in peritoneal tissues of PD patients with ultrafiltration failure, along with the presence of an enhanced inflammatory response. *In vitro* experiments showed that exposure to high glucose concentrations enhanced EP4 expression in rat peritoneal mesothelial cells (RPMCs). High-glucose–induced expression of inflammatory cytokines (monocyte chemoattractant protein-1, tumour necrosis factor α, and interleukin 1β) was significantly reduced in RPMCs treated with ONO-AE3-208, an EP4 receptor antagonist. ONO-AE3-208 also significantly decreased the expression of extracellular matrix proteins induced by high glucose concentrations. Furthermore, ONO-AE3-208 blunted activation of the NLR family pyrin domain containing 3 (NLRP3) inflammasome and phosphorylation of nuclear factor kappa B (NF-κB) (p-p65). To further investigate the functional role of EP4, ONO-AE3-208 was administrated for 4 weeks in a rat model of PD, the results of which showed that ONO-AE3-208 inhibited peritoneal fibrosis and improved peritoneal dysfunction. Additionally, inflammatory cytokines in the peritoneum of PD rats treated with ONO-AE3-208 were downregulated, in line with inhibition of the NLRP3 inflammasome and NF-κB phosphorylation. In conclusion, an EP4 antagonist reduced the development of peritoneal fibrosis, possibly by suppressing NLRP3 inflammasome- and p-p65–mediated inflammatory responses. Our findings suggest that an EP4 antagonist may be therapeutically beneficial for PD-associated peritoneal fibrosis.

## Introduction

Peritoneal dialysis (PD) is a well-established treatment option for patients with end-stage renal disease (ESRD). After continuous exposure to high-glucose PD fluid, histologic changes occur in the peritoneum, presenting as loss of the mesothelial monolayer, increased deposition of extracellular matrix, and angiogenesis (Zhou et al., 2016). Long-term infusion of bioincompatible PD fluid can lead to peritoneal fibrosis, eventually resulting in discontinuation of PD therapy (Fusshoeller, 2008; Morelle et al., 2015). Currently, no effective therapies are available to prevent the development of peritoneal fibrosis in clinical practice. Thus, there is an urgent need to develop a specific and effective therapy to prevent the development and progression of peritoneal fibrosis.

Several studies have demonstrated that chronic sterile inflammation induced by PD solutions with a high glucose content is involved in the pathogenesis of peritoneal fibrosis (Lai et al., 2007; Rosengren et al., 2013). Peritoneal injury caused by PD fluid leads to activation of nuclear factor kappa B (NF-κB); promotes the release of proinflammatory cytokines, including interleukin-6 (IL-6), IL-1β, tumour necrosis factor-α (TNF-α), and monocyte chemoattractant protein-1 (MCP-1); and increases the synthesis of extracellular matrix protein (Liu et al., 2015; Chu et al., 2017). Additionally, our recent study showed that microsomal prostaglandin E synthase-1 (mPGES-1), a catalytic enzyme for the production of prostaglandin E2 (PGE2), was induced in peritoneal mesothelial cells treated with high levels of glucose. We also found that mPGES-1 increased the synthesis of extracellular matrix proteins by activating the NLR family pyrin domain containing 3 (NLRP3) inflammasome (Luo et al., 2021). These findings suggest that targeting mPGES-1 may attenuate the progression of peritoneal fibrosis; however, the use of mPGES-1 inhibitors in clinical practice is limited because they also inhibit physiologic functions of PGE2.

Results from previous research and our recent study showed that PGE2 secretion was significantly elevated in peritoneal mesothelial cells treated with high levels of glucose (Sitter et al., 1998; Liu et al., 2007; Luo et al., 2021). PGE2 exerts its pharmacologic activity *via* four receptor subtypes: EP1, EP2, EP3, and EP4. EP1 regulates intracellular Ca^2+^ concentration, EP3 inhibits the intracellular increase in cyclic adenosine 3′,5′-monophosphate (cAMP) concentration, EP2, and EP4 increase intracellular levels of cAMP, and EP4 is involved in the phosphatidylinositol 3-kinase signal pathway by regulating NF-κB (Tang et al., 2012; Sheppe et al., 2018). Recent studies have shown that PGE2 participates in inflammation by acting on the EP4 receptor. For example, PGE2 stimulated the EP4 receptor to promote a chronic inflammatory response in a rat model of rheumatoid arthritis (Chen et al., 2010). Furthermore, PGE2 facilitated T helper type 1 (TH1) cell differentiation and amplified TH17 cell expansion *in vitro*, and administration of an EP4 antagonist in an animal model of experimental autoimmune encephalomyelitis decreased the accumulation of TH1 and TH17 cells and suppressed disease progression (Yao et al., 2009). However, the role of EP4 in modulating the inflammatory process during PD-associated peritoneal fibrosis remains unclear.

In this study, we examined EP4 expression in the peritoneum of PD patients with ultrafiltration failure (UFF) and rat peritoneal mesothelial cells (RPMCs) stimulated with high concentrations of glucose. Additionally, we evaluated the effects of ONO-AE3-208, a highly selective and potent functional EP4 receptor antagonist, on pathologic changes associated with fibrosis *in vitro* and *in vivo*. We also determined the mechanisms by which EP4 receptor inhibition reduces the progression of peritoneal fibrosis.

## Materials and methods

### Reagents

ONO-AE3-208 was purchased from Cayman Chemistry (Michigan, United States). We obtained 4.25% PD fluid from Baxter Healthcare (Guangzhou, China). The sources for the primary and secondary antibodies used in this study were as follows: rabbit polyclonal anti-EP4 (no. 101775) was obtained from Cayman Chemistry; rabbit monoclonal anti-fibronectin (no. ab45688), rabbit monoclonal anti-collagen I (no. ab138492), rabbit monoclonal anti-NLRP3 (no. ab263899), rabbit monoclonal anti–caspase-1 (no. ab179515), rabbit monoclonal anti-vimentin (no. ab92547), goat anti-rabbit IgG H&L Alexa Fluor 488-conjugated secondary antibody (no. ab150077), and goat anti-mouse IgG H&L Alexa Fluor 647-conjugated secondary antibody (no. ab150115) were obtained from Abcam (Cambridge, MA, United States); and mouse monoclonal anti-E-cadherin (no. 14472), rabbit monoclonal anti-phospho-NF-κB (p-p65, no. 3033), rabbit monoclonal anti-NF-κB (p65, no. 8242), β-actin (no.3700), horseradish peroxidase (HRP)-conjugated anti-mouse IgG (no. 7076), and HRP-conjugated anti-rabbit IgG (no.7074) were obtained from Cell Signaling Technology (Danvers, MA, United States). IL-1β, IL-18, TNF-α, and MCP-1 enzyme-linked immunosorbent assay (ELISA) kits were purchased from Dakewe Biotech (Shenzhen, China). The PrimeScript™ RT Reagent Kit (no. RR470A) and the SYBR Green PCR Kit (no. RR820A) were purchased from TaKaRa (Japan). The DAB Kit (no. GK500710) was purchased from Gene Tech (China).

### Human sample collection

Peritoneal tissues and nocturnal peritoneal dialysate samples were collected from ESRD patients, new-onset PD patients and PD patients with UFF at Shunde Hospital of Southern Medical University. New-onset PD patients were defined as patients who had started PD therapy within the previous 6 months. UFF was defined as failing to achieve at least 400 ml of net ultrafiltration in a 4-h dwell period with 4.25% PD fluid (Teitelbaum, 2015). Peritoneal tissues were collected at the time of laparoscopy for PD catheter implantation or withdrawal. The peritoneal dialysate was drained after at least 8 h of dwell exchange.

Patients were excluded if they refused to provide written informed consent; had a malignancy, diabetes mellitus, or heterotopia endometriosis; or received nonselective non-steroidal anti-inflammatory drugs or cyclooxygenase 2 (COX-2)-selective inhibitors (e.g., celecoxib, nimesulide, meloxicam, rofecoxib) in the past 2 weeks. All patients provided written informed consent. The use of human biopsy and dialysate samples were approved by the Ethical Review Board of Shunde Hospital of Southern Medical University.

### Cell culture and treatment

RPMCs were cultured at 37°C with 5% carbon dioxide in Dulbecco’s Modified Eagle Medium/Nutrient Mixture F-12 medium containing 10% fetal bovine serum (FBS, Gibco, United States), 1% penicillin, and 1% streptomycin. RPMCs were treated with the following glucose concentrations: 5.5 mmol/L, 83.3 mmol/L, 138 mmol/L, and 234.6 mmol/L. The 83.3 mmol/L, 138 mmol/L, and 234.6 mmol/L concentrations represent the glucose concentrations in 1.5%, 2.5%, and 4.25% PD fluid. Cell Counting Kit-8 (CCK-8, Solarbio, China) was used to assess the effects of different glucose concentrations on cell viability. To examine the expression of EP4 in RPMCs, cells were first deprived of serum for 24 h and then treated with d-glucose at high (138 mmol/L) concentration for 0, 24, 48, and 72 h. RPMCs were also treated with 138 mmol/L mannitol. RPMCs were treated with different concentrations of ONO-AE3-208 for 24 h, and then CCK-8 was used to determine cell viability. To examine the effects of ONO-AE3-208 on high-glucose–induced inflammation and fibrosis, RPMCs were deprived of serum for 24 h and then exposed to 138 mmol/L glucose in the presence of ONO-AE3-208 (100 and 200 nM). After exposure for 24 or 48 h, the cells were harvested for quantitative reverse-transcription polymerase chain reaction testing (RT-PCR) or western blotting. All *in vitro* experiments were repeated at least thrice.

### Animal models and experimental design

Male Sprague–Dawley rats weighing 200–220 g were purchased from the Guangdong Medical Laboratory Animal Center (Guangdong, China). A rat model of peritoneal fibrosis was created by intraperitoneal injection of 4.25% PD fluid at a dose of 100 ml/kg daily for 4 weeks (Zhou et al., 2013). To investigate the effects of ONO-AE3-208 on peritoneal fibrosis, rats were intraperitoneally injected with ONO-AE3-208 at 0.2 mg/kg per day (Wang et al., 2014). Rats were randomly divided into three groups: 1) rats injected intraperitoneally with 100 ml/kg saline (control group; *n* = 6); 2) rats injected intraperitoneally with 100 ml/kg PD fluid (PD group; *n* = 6); and 3) rats injected intraperitoneally with 100 ml/kg PD fluid + ONO-AE3-208 (PD + ONO-AE3-208 group; *n* = 6).

After 28 days of PD, a peritoneal equilibrium test was performed on each rat before they were killed. Briefly, 100 ml/kg of 4.25% PD fluid was injected intraperitoneally, and dialysate and blood samples were collected at 0 and 4 h of dwell time. Parietal and visceral peritoneal tissues were collected for further analysis. The animal protocol was reviewed and approved by the Animal Experimental Ethics Committee at Southern Medical University.

### Analysis of peritoneal permeability

Concentrations of glucose and creatinine in the dialysate and plasma were assessed using an automatic biochemical instrument (AU5800; Beckman Coulter, California, United States). Absorption of glucose from the dialysate (D/D0) and the dialysate-to-plasma ratio of creatinine (D/P_Scr_) were used to determine peritoneal permeability.

### Histology and immunohistochemistry

Changes in peritoneal tissues were assessed by haematoxylin and eosin staining or Masson’s trichrome staining of paraformaldehyde-fixed, paraffin-embedded 4-μm–thick sections. Peritoneum thickness was expressed as the mean of five independent measurements for each section. A microwave-based antigen retrieval technique was used to examine the immunohistochemistry results, as previously described (Luo et al., 2021). Briefly, 4-µm–thick sections were de-paraffinised, rehydrated, and incubated in hydrogen peroxide to block endogenous peroxidase, then antigen retrieval was performed. The signal was visualised using a DAB kit, and slides were viewed with a Leica microscope equipped with a digital camera.

### Western blotting

Western blotting analysis of peritoneal tissues and cell samples was conducted as previously described (Luo et al., 2021). The densitometry analysis was performed using the ImageJ software.

### RNA extraction and quantitative RT-PCR

Total RNA was extracted from the peritoneal tissues and cell samples using Trizol reagent. cDNA was synthesised using the PrimeScript™ RT Reagent Kit according to the manufacturer’s protocol. Real-time PCR was performed using the SYBR Green PCR Kit according to the manufacturer’s instructions. Primers used in this study are listed in Table 1. Levels of mRNAs were normalised to β-actin in each sample, and data were presented as the relative fold change.

**TABLE 1:** Primer sequences for q-PCR.

Gene symbol	Primer sequences
β-actin	5′-TGT​GAC​GTT​GAC​ATC​CGT​AAA​G-3′
	5′-GGC​AGT​AAT​CTC​CTT​CTG​CAT​C-3′
IL-1β	5′-CTA​TGG​CAA​CTG​TCC​CTG​AA-3′
	5′-GGC​TTG​GAA​GCA​ATC​CTT​AAT​C-3′
TNF-α	5′-CGT​GTT​CAT​CCG​TTC​TCT​ACC-3′
	5′-CAG​AGC​CAC​AAT​TCC​CTT​TCT​A-3′
MCP-1	5′-GAT​CTC​TCT​TCC​TCC​ACC​ACT​A-3′
	5′-TTA​ACT​GCA​TCT​GGC​TGA​GAC-3
Fibronectin	5′-ACC​GAA​ATC​ACA​GCC​AGT​AG-3
	5′-CCT​CCT​CAC​TCA​GCT​CAT​ATT​C-3
Collagen Ⅰ	5′-CCT​GTC​TGC​TTC​CTG​TAA​ACT​C-3
	5′-GTT​CAG​TTT​GGG​TTG​CTT​GTC -3
Vimentin	5′- GTC​CGT​GTC​CTC​GTC​CTC​CTA​C -3
	5′- AGG​TGC​GGG​TGG​ATG​TGG​TC -3

### Immunofluorescence staining

Two-colour immunofluorescence staining was performed on the paraformaldehyde-fixed, paraffin-embedded sections. Sections were de-paraffinised, rehydrated, and antigen repaired. Sections were incubated with blocking buffer (3% bovine serum albumin [BSA] in phosphate-buffered saline [PBS]). Peritoneal tissues were incubated overnight at 4°C with polyclonal rabbit anti-EP4 (1:100) and monoclonal mouse anti-E-cadherin (1:100), followed by incubation with goat anti-rabbit IgG (Alexa Fluor 488, 1:1,000) and goat anti-mouse IgG (Alexa Fluor 647, 1:1,000) for 1 h at room temperature. Nuclei were counterstained with 4′,6-diamidino-2-phenylindole (DAPI). Positive staining was measured using a fluorescent microscope (Leica, Germany).

Cells were cultured on glass coverslips and fixed with methanol for 10 min at room temperature. They were then washed with PBS and permeabilised in 0.1% Triton-X for 10 min at room temperature, followed by incubation in blocking buffer (5% BSA in PBS) for 1 h at room temperature. The cells were subsequently incubated with monoclonal rabbit anti–phospho-NF-κB (1:100) at 4°C overnight, and then incubated with Alexa Fluor 488-conjugated anti-rabbit IgG (1:1,000) antibody for 1 h at room temperature, followed by staining with DAPI. Positive staining was measured using a fluorescent microscope (Leica, Germany).

### ELISA

ELISA detection of IL-1β, IL-18, TNF-α, and MCP-1 proteins in the dialysate was performed according to the manufacturer’s instructions.

### Statistical analysis

**FIGURE 1:**
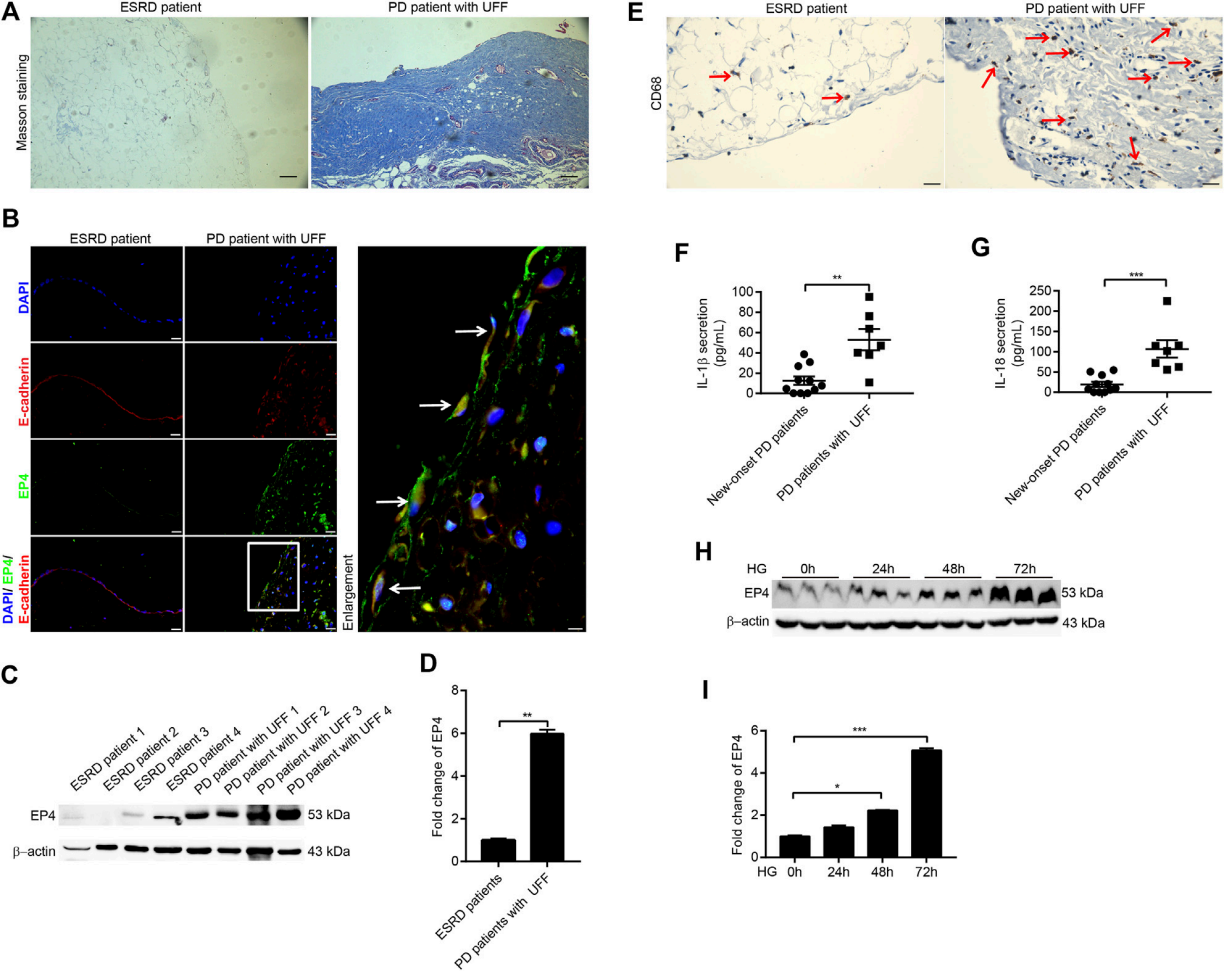
Expression of EP4 increased during peritoneal fibrosis in patients with UFF and high-glucose–induced RPMCs. **(A)** Masson staining from peritoneal tissues of ESRD patient and PD patient with UFF. Scale bar = 100 μm. **(B)** Immunofluorescence microscopy of peritoneal tissue sections stained for EP4 (green) and E-cadherin (red) with DAPI (blue). Arrows represent EP4- and E-cadherin–positive mesothelial cells. Scale bar = 25 μm. **(C)** Peritoneal tissue lysates from ESRD patients and PD patients with UFF were subjected to western blotting analysis with specific antibodies against EP4 and β-actin. **(D)** EP4 expression levels were quantified by densitometry, normalised with β-actin, and presented as fold changes. **(E)** Immunohistochemistry analysis of peritoneal tissue sections stained for CD68. Arrows represent CD68-positive cells. Scale bar = 25 μm. **(F,G)** Dialysates from new-onset PD patients (*n* = 11) and PD patients with UFF (*n* = 7) were subjected to ELISA analysis to detect secretion of IL-1β **(F)** and IL-18 **(G)**. **(H)** Cell lysates from RPMCs exposed to 138 mmol/L glucose at different times were subjected to western blotting analysis with specific antibodies against EP4 and β-actin. **(I)** EP4 expression levels were quantified by densitometry, normalised with β-actin, and presented as fold changes. Data are presented as mean ± SEM. ^*^ *p* < 0.05, ^**^ *p* < 0.01, ^***^ *p* < 0.001.

**FIGURE 2:**
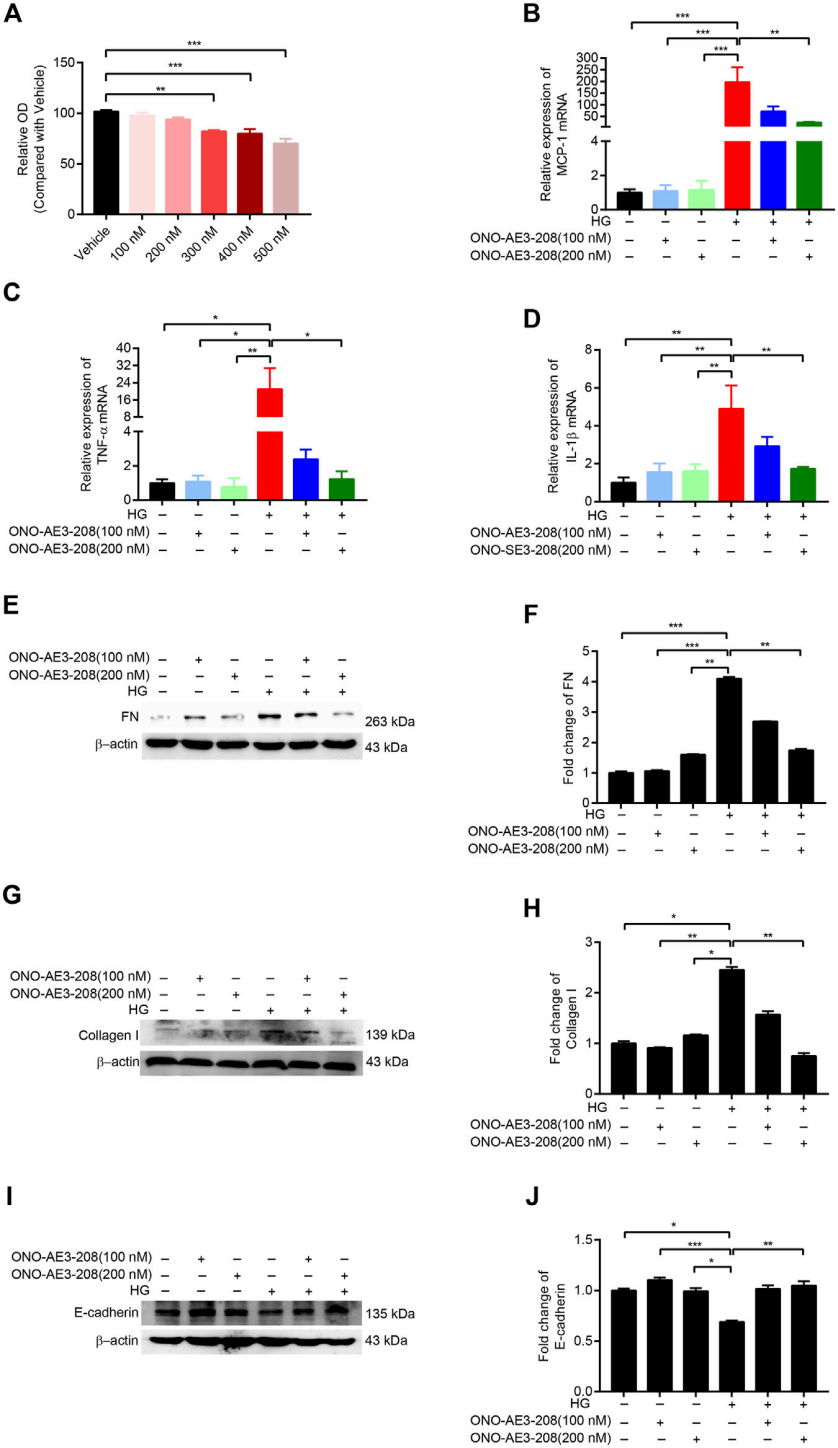
ONO-AE3-208 decreased inflammatory activity and synthesis of extracellular matrix in cultured RPMCs. **(A)** Cell viability from RPMCs treated with different doses of ONO-AE3-208 was examined by CCK-8. **(B–D)** Real-time quantitative PCR analysis of MCP-1, TNF-α, and IL-1β levels in RPMCs exposed to different doses of ONO-AE3-208 or 138 mmol/L glucose. **(E,G,I)** Cell lysates from RPMCs treated with different doses of ONO-AE3-208 or 138 mmol/L glucose were subjected to western blotting analysis with specific antibodies against fibronectin (FN), collagen I, E-cadherin, and β-actin. **(F,H,J)** Expression levels of FN, collagen I, and E-cadherin were quantified by densitometry, normalised with β-actin, and presented as fold changes. Data are presented as mean ± SEM. ^*^ *p* < 0.05, ^**^ *p* < 0.01, ^***^ *p* < 0.001.

**FIGURE 3:**
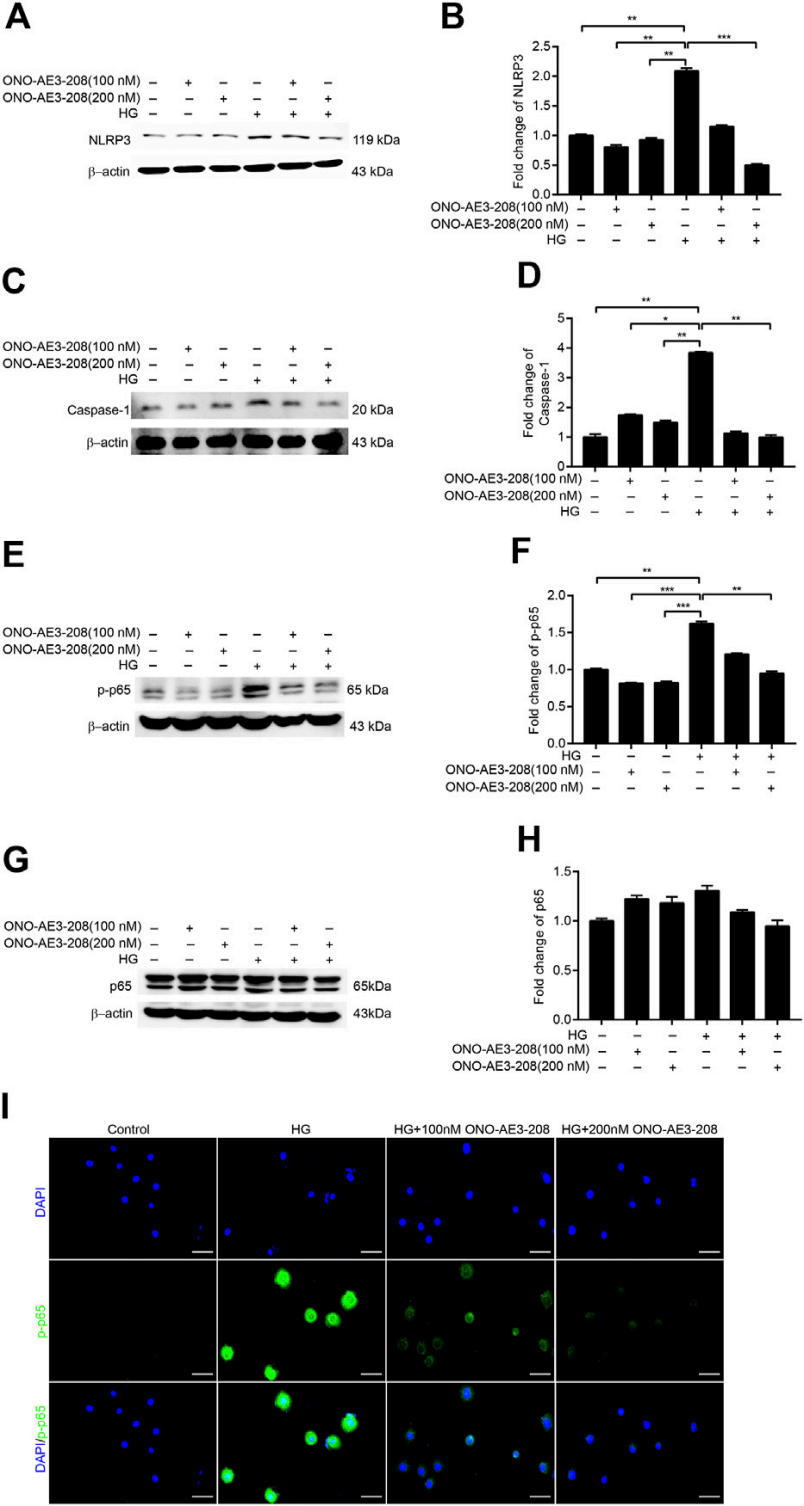
ONO-AE3-208 inhibited activation of the NRLP3 inflammasome and phosphorylation of NF-κB in cultured RPMCs. **(A,C)** Cell lysates from RPMCs treated with different doses of ONO-AE3-208 or 138 mmol/L glucose were subjected to western blotting analysis with specific antibodies against NLRP3, caspase-1, and β-actin. **(B,D)** Expression levels of NLRP3 and caspase-1 were quantified by densitometry, normalised with β-actin, and presented as fold changes. **(E,G)** Cell lysates from RPMCs treated with different doses of ONO-AE3-208 or 138 mmol/L glucose were subjected to western blotting analysis with specific antibodies against p-p65, p65, and β-actin. **(F,H)** Expression levels of p-p65 and p65 were quantified by densitometry, normalised with β-actin, and presented as fold changes. **(I)** Photomicrographs showing immunofluorescence staining of p-p65 and DAPI in RPMCs exposed to different doses of ONO-AE3-208 or 138 mmol/L glucose. Scale bar = 50 μm. Data are represented as mean ± SEM. ^*^ *p* < 0.05, ^**^ *p* < 0.01, ^***^ *p* < 0.001.

All data are presented as mean ± standard error of the mean (SEM). Significant differences between two groups were assessed using unpaired, two-tailed Student’s t-test. Multiple groups were compared using one-way analysis of variance (ANOVA), followed by Dunnett’s multiple comparisons test *versus* the high-glucose group (Figures 2B–D,F,H,J, 3B,D,F,H) or *versus* the high-glucose 0-h group, vehicle group, 5.5 mmol/L glucose group, or mannitol 0-h group (Figures 1I, 2A, Supplementary Figures 1A,C). For *in vivo* data, statistical differences were assessed using one-way ANOVA, followed by comparisons of the mean of each column with the mean of every other column. *p* < 0.05 was deemed indicative of statistical significance. Statistical and data analyses were conducted using GraphPad Prism 7.

## Results

### Expression of EP4 increased during peritoneal fibrosis in patients with UFF and high-glucose–induced RPMCs

We first evaluated the expression of EP4 in the peritoneal tissues of ESRD patients and PD patients with UFF. On Masson’s trichrome staining, the peritoneal tissues of ESRD patients exhibited no substantial peritoneal fibrosis, whereas the peritoneal tissues of PD patients with UFF showed severe peritoneal fibrosis, with increased accumulation of extracellular matrix (Figure 1A). Double indirect immunofluorescent staining for EP4 and E-cadherin showed partial loss of E-cadherin and increased EP4 expression in the peritoneum of PD patients with UFF, and the EP4 staining co-localized with the E-cadherin staining (Figure 1B). As E-cadherin is a mesothelial cell marker, these results indicate that EP4 was expressed in the residual mesothelial cells in PD patients with UFF.

Next, 4 peritoneal tissues from ESRD patients and 4 peritoneal tissues from PD patients with UFF were used for western blotting to investigate EP4 protein expression. The western blotting results showed that EP4 expression was significantly increased in the peritoneal tissues of PD patients with UFF (Figures 1C,D). We also found that CD68-positive cells were increased in the peritoneum of PD patients with UFF, whereas these cells were rarely found in the peritoneum of ESRD patients (Figure 1E). Dialysate concentrations of IL-1β and IL-18 of new-onset PD patients and PD patients with UFF were also analyzed. As shown in Figures 1F,G, levels of both IL-1β and IL-18 were significantly increased in PD patients with UFF. These results suggest that an increased inflammatory response was observed in the peritoneum of PD patients with UFF.

We also investigated changes in EP4 expression in RPMCs after damage induced by high levels of glucose. CCK-8 testing showed that cell viability was not affected by a glucose concentration of 138 mmol/L (Supplementary Figure 1A). After exposing RPMCs to 138 mmol/L glucose for 0, 24, 48, and 72 h, EP4 expression was significantly increased in these high-glucose–induced RPMCs (Figures 1H,I). By contrast, 138 mmol/L mannitol did not increase EP4 expression (Supplementary Figures 1B,C). Taken together, these results indicate that there is an increased inflammatory response in the peritoneum of PD patients with UFF and that EP4 is upregulated in the peritoneum of PD patients with UFF and RPMCs exposed to high glucose levels.

### ONO-AE3-208 decreased inflammatory activity and synthesis of extracellular matrix in cultured RPMCs

Cultured RPMCs were treated with varying concentrations of ONO-AE3-208. CCK-8 results showed that 100 nM and 200 nM concentrations of ONO-AE3-208 had no significant effect on cell viability (Figure 2A). We then incubated RPMCs with 138 mmol/L glucose and 100 nM or 200 nM ONO-AE3-208. Quantitative RT-PCR assay showed that mRNA levels of MCP-1, TNF-α, and IL-1β were significantly increased in response to high glucose, and ONO-AE3-208 treatment inhibited this expression (Figures 2B–D). Exposure of RPMCs to 138 mmol/L glucose increased the expression of fibronectin (FN) and collagen I and decreased the expression of E-cadherin; treatment with ONO-AE3-208 inhibited this high-glucose–induced upregulation of FN and collagen I and downregulation of E-cadherin (Figures 2E–J). These results demonstrate the inhibitory effects of ONO-AE3-208 on inflammatory activity and extracellular matrix synthesis *in vitro*.

### ONO-AE3-208 inhibited activation of NLRP3 inflammasome and phosphorylation of NF-κB in cultured RPMCs

Our previous study showed that mPGES-1 contributed to peritoneal fibrosis by activating the NLRP3 inflammasome (Luo et al., 2021); thus, in this study, we examined the effects of ONO-AE3-208 on NLRP3 inflammasome activation. NLRP3 and caspase-1 activation were induced in cultured RPMCs treated with 138 mmol/L glucose, a phenomenon that was markedly blocked by treatment with ONO-AE3-208 (Figures 3A–D).

As NF-κB–induced inflammatory signalling pathways play a pivotal role in peritoneal fibrosis (Shi et al., 2021), we also explored whether the anti-inflammatory effects of ONO-AE3-208 were mediated by changes in phosphorylated NF-κB. We found that phosphorylated NF-κB (p-p65) was substantially increased in cultured RPMCs stimulated with 138 mmol/L glucose, and that ONO-AE3-208 downregulated phosphorylated NF-κB (p-p65) but had no significant effect on total NF-κB (p-65) (Figures 3E–H). Additionally, immunofluorescence staining revealed increased nuclear translocation of phosphorylated NF-κB (p-p65) in cultured RPMCs treated with high glucose, whereas ONO-AE3-208 abrogated high-glucose–induced translocation of phosphorylated NF-κB (p-p65) (Figure 3I). These data suggest that ONO-AE3-208 regulates activation of the NLRP3 inflammasome and phosphorylation of NF-κB *in vitro*.

### Administration of ONO-AE3-208 reduced peritoneal pathologic injury and improved peritoneal dysfunction in a rat model of peritoneal dialysis

**FIGURE 4:**
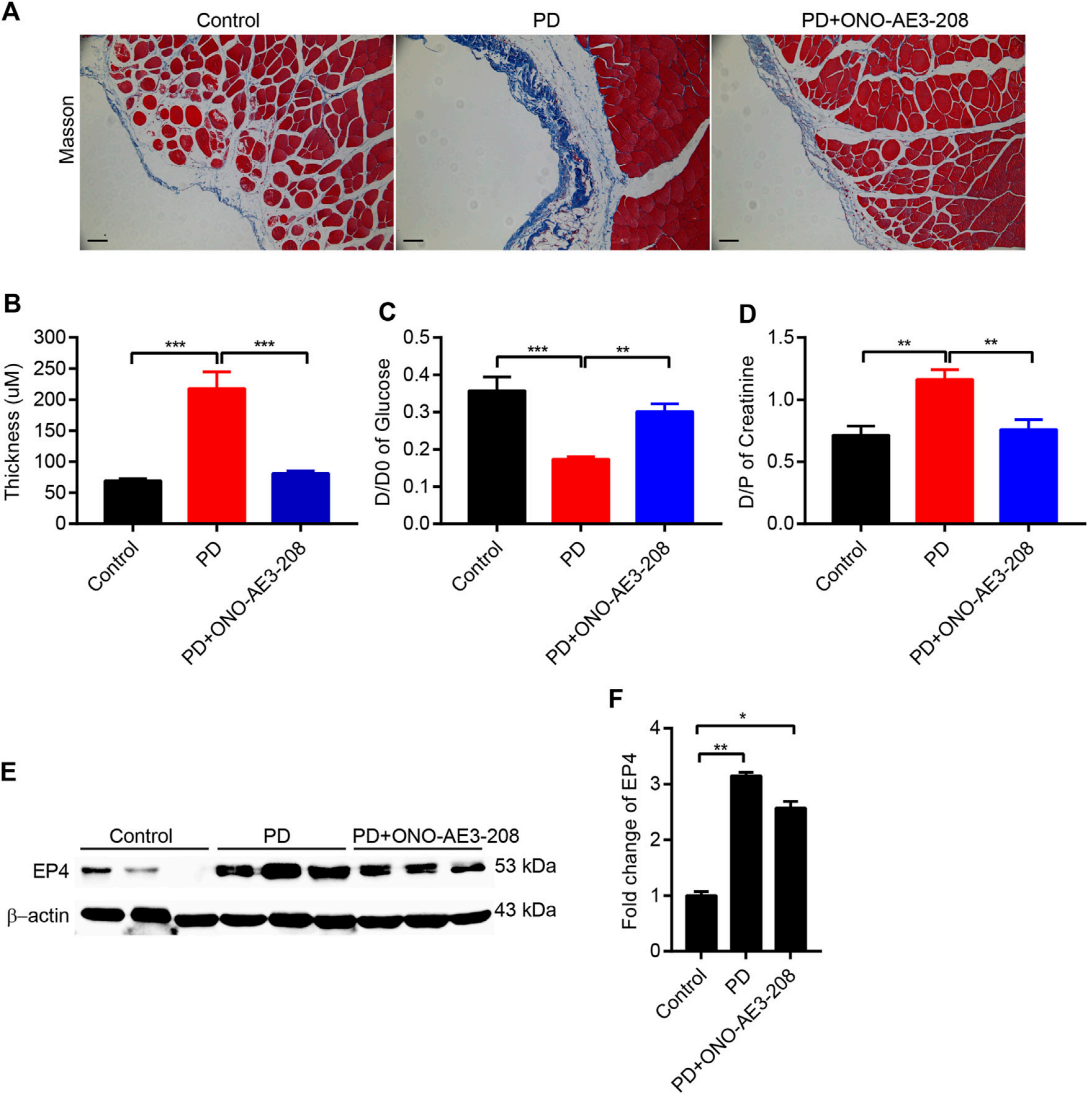
Administration of ONO-AE3-208 reduced peritoneal pathologic injury and improved peritoneal dysfunction in a rat model of peritoneal fibrosis. **(A)** Photomicrographs illustrating Masson staining of the parietal peritoneum in each animal group. **(B)** Thickness of the peritoneal membrane in each group. **(C,D)** Peritoneal function was assessed by the transfer of glucose (D/D0) and creatinine (D/P_Scr_). **(E)** Peritoneal tissue lysates from each group were subjected to western blotting analysis with specific antibodies against EP4 and β-actin. **(F)** Expression levels of EP4 were quantified by densitometry, normalised with β-actin, and presented as fold changes. Data are presented as mean ± SEM. ^*^ *p* < 0.05, ^**^ *p* < 0.01, ^***^ *p* < 0.001.

To further elucidate the role of ONO-AE3-208 in the development of peritoneal fibrosis and functional injury in response to chronic PD fluid infusion, we established a rat model of peritoneal fibrosis and concurrently treated the animals with ONO-AE3-208. Masson’s trichrome staining showed that compared with the peritoneal tissues of control rats, those in the PD group exhibited loss of the mesothelial cell monolayer and increased thickness of the submesothelial compact zone (Figures 4A,B). These histologic changes were accompanied by increased peritoneal transport of glucose (D/D0) and increased peritoneal transport of creatinine (D/P_Scr_) (Figures 4C,D), indicating increased peritoneal permeability in PD rats. In contrast, administering ONO-AE3-208 prevented peritoneal thickening and improved peritoneal transport function, as indicated by a decreased D/D0 and decreased D/P_Scr_ (Figures 4A–D). We also evaluated EP4 expression in the peritoneum *in vivo*. Upregulation of EP4 in the fibrotic peritoneal tissues was confirmed, and ONO-AE3-208 had little effect on EP4 expression (Figures 4E,F). These results indicate that ONO-AE3-208 can alleviate peritoneal pathologic injury and improve peritoneal dysfunction *in vivo*.

### Administration of ONO-AE3-208 prevented inflammation in a rat model of peritoneal dialysis

**FIGURE 5:**
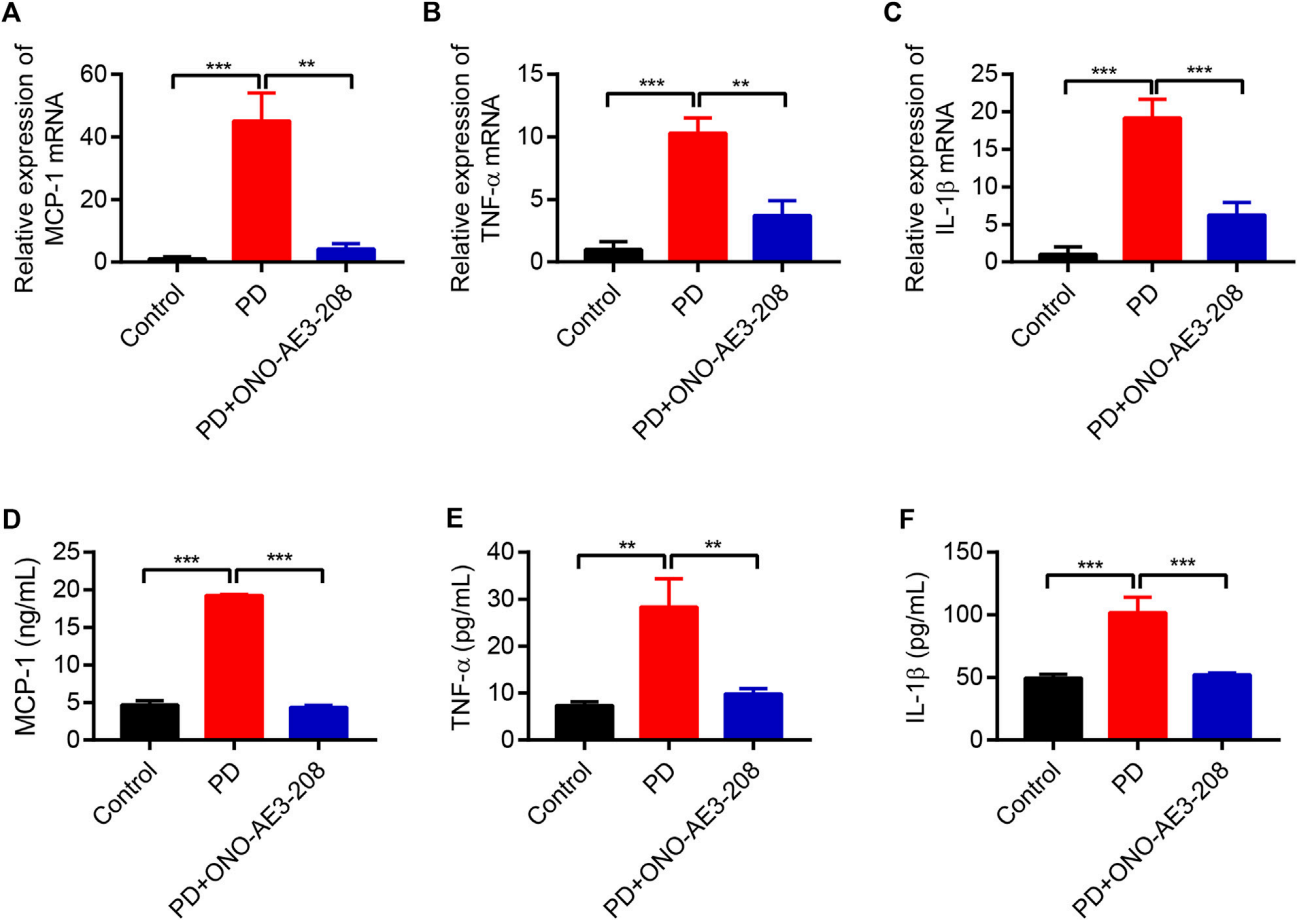
Administration of ONO-AE3-208 prevented inflammation in a rat model of peritoneal fibrosis. **(A–C)** Real-time quantitative PCR analysis of MCP-1, TNF-α, and IL-1β levels in the visceral peritoneum for each animal group. **(D–F)** ELISA results of the secretion of MCP-1, TNF-α, and IL-1β in the dialysate for each group. Data are presented as mean ± SEM. ^**^ *p* < 0.01, ^***^ *p* < 0.001.

Based on the results of our *in vitro* study, we further evaluated the effects of ONO-AE3-208 on inflammatory cytokine expression *in vivo*. Levels of MCP-1, TNF-α, and IL-1β mRNA in the visceral peritoneum increased in rats receiving 4.25% PD fluid, and these levels decreased after treatment with ONO-AE3-208 (Figures 5A–C). Secretion of these inflammatory cytokines in the PD fluid was further evaluated using ELISA. Injection of 4.25% PD fluid significantly increased MCP-1, TNF-α, and IL-1β concentrations in the effluent, and treatment with ONO-AE3-208 effectively reduced their secretion (Figures 5D–F). These results demonstrate that ONO-AE3-208 inhibits peritoneal inflammation during PD *in vivo*.

### Administration of ONO-AE3-208 prevented peritoneal fibrosis in a rat model of peritoneal dialysis

**FIGURE 6:**
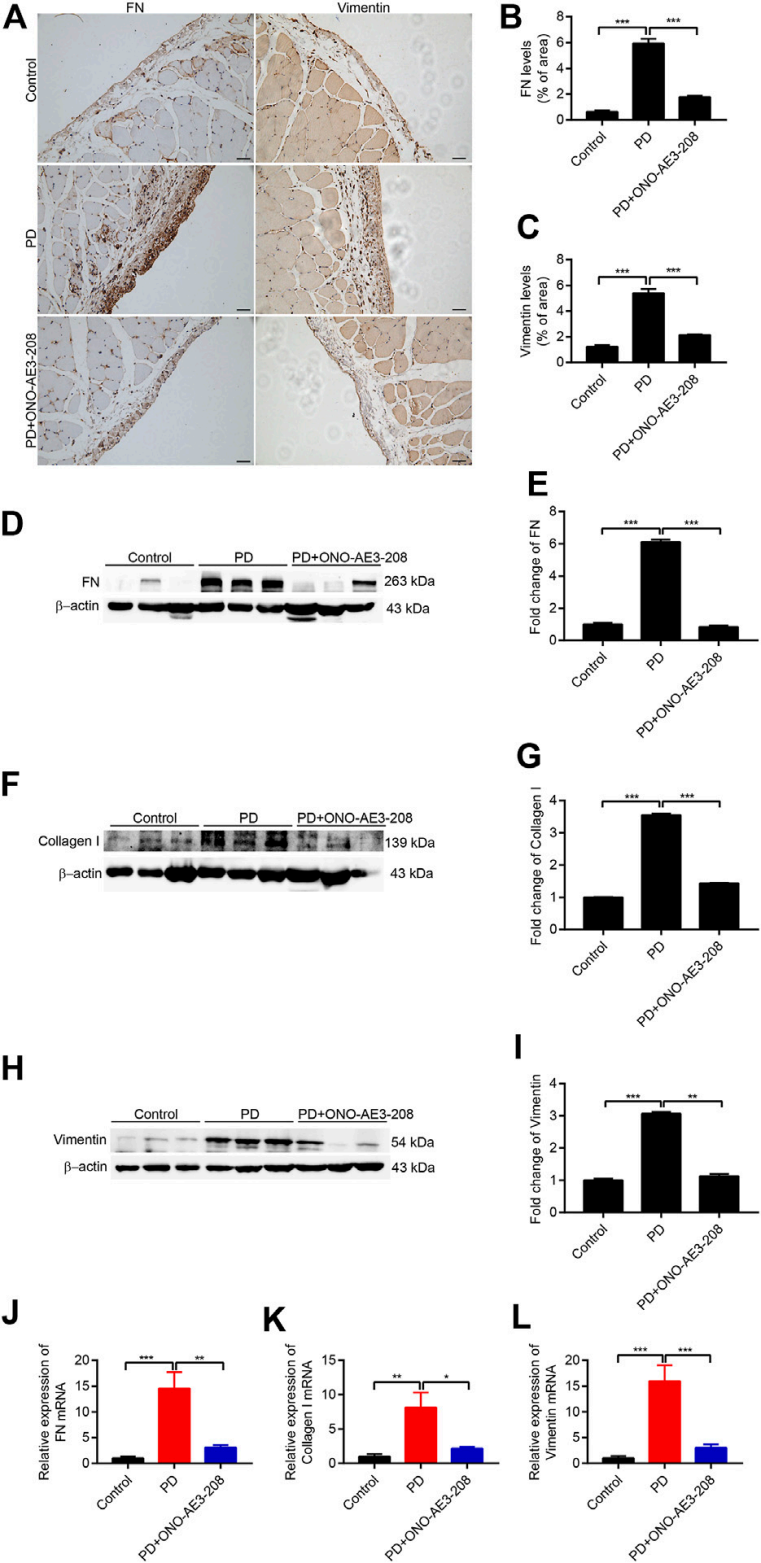
Administration of ONO-AE3-208 prevented peritoneal fibrosis in a rat model of peritoneal fibrosis. **(A)** Photomicrographs showing immunohistochemistry staining of FN and vimentin from peritoneal tissues in each group. Scale bar = 50 μm. **(B,C)** Areas positive for FN and vimentin. **(D,F,H)** Peritoneal tissue lysates from each animal group were subjected to western blotting analysis with specific antibodies against FN, collagen I, vimentin, and β-actin. **(E,G,I)** Expression levels of FN, collagen I, and vimentin were quantified by densitometry, normalised with β-actin, and presented as fold changes. **(J–L)** Real-time quantitative PCR analysis of FN, collagen I, and vimentin levels in the visceral peritoneum for each group. Data are presented as mean ± SEM. ^*^ *p* < 0.05, ^**^ *p* < 0.01, ^***^ *p* < 0.001.

We next examined the preventive effects of ONO-AE3-208 on peritoneal fibrosis in response to long-term PD fluid infusion. Results of immunohistochemical staining demonstrated that ONO-AE3-208 inhibited the formation of FN and vimentin and significantly reduced FN- and vimentin-positive areas (Figures 6A–C). The protective effects of ONO-AE3-208 on peritoneal fibrosis were further demonstrated by its ability to inhibit FN, collagen I, and vimentin at both the mRNA and protein levels (Figures 6D–L). These data thereby suggest that ONO-AE3-208 prevents peritoneal fibrosis *in vivo*.

### Administration of ONO-AE3-208 inhibited NLRP3 inflammasome activation and NF-κB phosphorylation in a rat model of peritoneal dialysis

**FIGURE 7:**
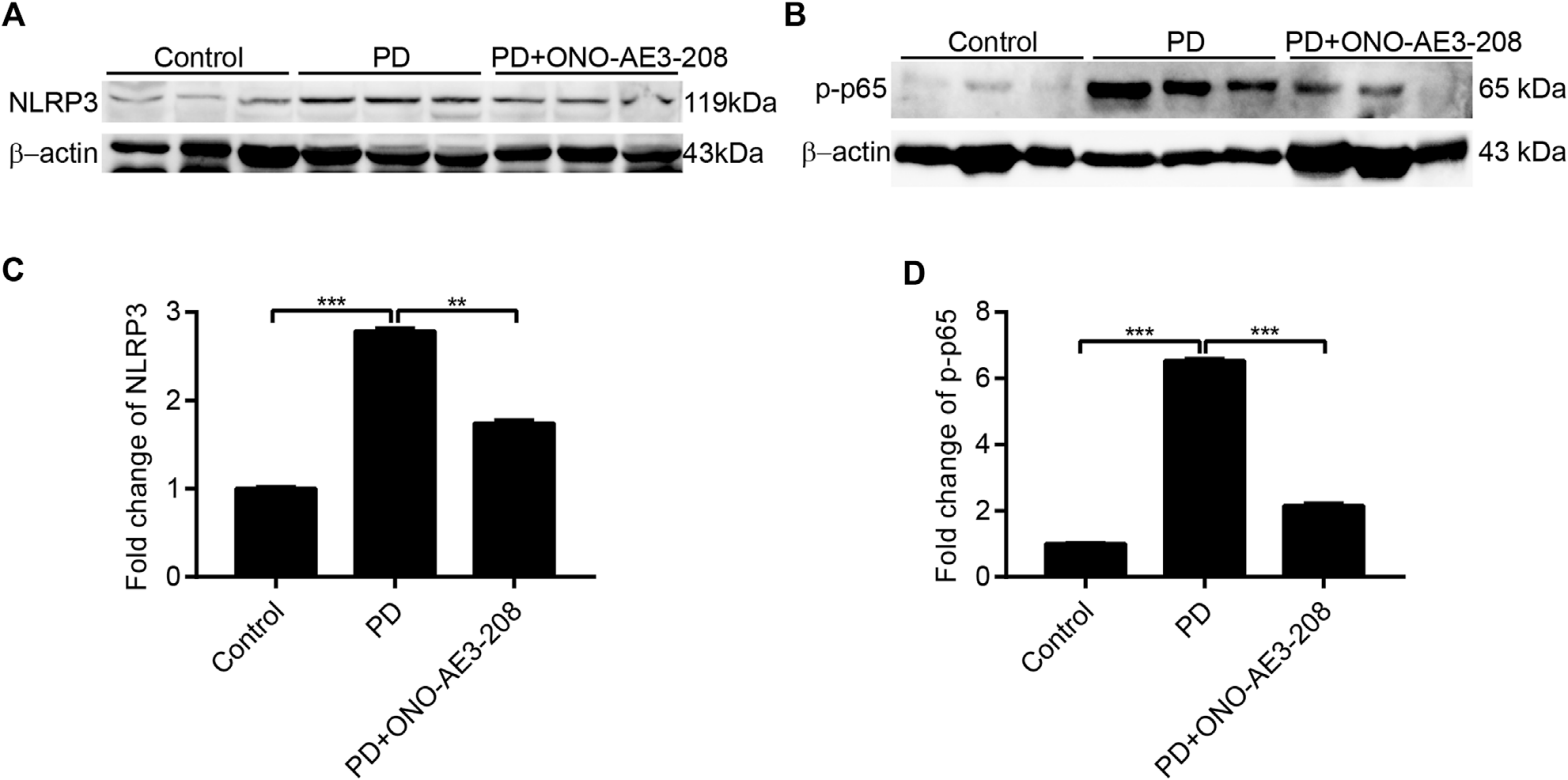
Administration of ONO-AE3-208 inhibited NLRP3 inflammasome activation and NF-κB phosphorylation in a rat model of peritoneal dialysis. **(A,B)** Peritoneal tissue lysates from each animal group were subjected to western blotting analysis with specific antibodies against NLRP3, p-p65, and β-actin. **(C,D)** Expression levels of NLRP3 and p-p65 were quantified by densitometry, normalised with β-actin, and presented as fold changes. Data are presented as mean ± SEM. ^**^ *p* < 0.01, ^***^ *p* < 0.001.

Finally, we investigated the effects of ONO-AE3-208 on activation of the NLRP3 inflammasome and NF-κB in peritoneal tissues *in vivo*. Western blotting analysis revealed that NLRP3 and p-p65 expression were significantly induced in the peritoneum of PD rats and significantly inhibited in the peritoneal tissues of rats treated with ONO-AE3-208 (Figures 7A–D). These data indicate that ONO-AE3-208 inhibits activation of the NLRP3 inflammasome and phosphorylation of NF-κB *in vivo*.

## Discussion

PGE2 is the major product of COX-2 and mPGES-1, and these two enzymes were previously reported to be increased in the peritoneum of a mouse model of PD and PD patients with UFF (Aroeira et al., 2009; Luo et al., 2021). PGE2 can bind to different EP receptors (EP1 to EP4), which are expressed in different cells and have opposing effects; therefore, blocking PGE2 using COX-2 or mPGES-1 inhibitors is clinically problematic. Targeting an EP receptor that specifically regulates the deleterious effects of COX-2/mPGES-1/PGE2 would be beneficial for preventing or treating peritoneal fibrosis. In this study, we found that PD-related peritoneal fibrosis was associated with increased expression of EP4 and that administration of an EP4 receptor antagonist (ONO-AE3-208) prevented progressive peritoneal fibrosis and functional injury of the peritoneal membrane. These results suggest that EP4 may be a potential therapeutic target for PD-associated peritoneal fibrosis.

Previous studies showed that EP4 was expressed in the brain, thymus, heart, lung, uterus, and kidney tissues of mice (Sugimoto and Narumiya, 2007; Aringer et al., 2018). Our study demonstrated that EP4 is also distributed in human peritoneal tissues. Peritoneal fibrosis is a common complication in patients undergoing long-term PD, and our findings of enhanced EP4 expression in the peritoneum of PD patients with UFF and RPMCs stimulated with high levels of glucose suggest that EP4 may play an important role in the development of this complication. Chronic inflammation promotes the development of peritoneal fibrosis. Our results are consistent with previous reports of the EP4 receptor playing a key role in a variety of disease models associated with increased inflammation, including type 1 diabetes (Rahman et al., 2018), endometritis (Li et al., 2019), angiotensin II-induced abdominal aortic aneurysm (Hiromi et al., 2020), airway inflammation (van Geffen et al., 2021), and nephrotoxic serum nephritis (Aringer et al., 2018).

The current study adds new evidence in support of the important role of EP4 in promoting inflammation, as indicated by the findings that EP4 blockade suppressed the expression of inflammatory cytokines in RPMCs in response to high glucose levels and in the peritoneal tissues of a rat model of PD. However, the role of EP4 in inflammation varies. In a mouse model of inflammatory bowel disease, PGE2 signalling repressed intestinal epithelial cell necroptosis and induced resolution of colitis through the EP4 receptor (Patankar et al., 2021). In addition, blockade of the EP4 receptor in a mouse model of autosomal dominant polycystic kidney disease resulted in more severe cystic disease and increased macrophage infiltration in the kidney tissue (Lannoy et al., 2020). Thus, functions of the EP4 receptor appear to be cell type- and context-dependent, and the differing effects on inflammation may be attributed to different disease models.

Emerging evidence shows that targeting the EP4 receptor has clear effects in various animal models of fibrosis. A specific EP4 antagonist significantly inhibited the autophagy of M2 macrophage-mediated hepatic stellate cells and improved liver fibrosis (Cao et al., 2022). Chronic repeated administration of an EP4 receptor-selective antagonist significantly attenuated the development of glomerulosclerosis and tubulointerstitial fibrosis in 5/6 nephrectomised rats (Mizukami et al., 2019). Administration of a PGE2 receptor EP4-selective agonist increased TNF-α (an inflammatory cytokine) levels and exacerbated renal fibrosis in an animal model of type 1 diabetes (Mohamed et al., 2013). Our results showed that the EP4 antagonist ONO-AE3-208 had beneficial effects in an experimental model of PD-associated peritoneal fibrosis. This was further demonstrated by the findings that ONO-AE3-208 protected against PD-induced peritoneal fibrosis and functional impairment of the peritoneal membrane, thus suggesting that ONO-AE3-208 may be a potential therapeutic agent for peritoneal fibrosis.

ONO-AE3-208 is a well-characterized EP4 receptor antagonist, which binds to the EP4 receptor, thereby blocking the interaction of PGE2 with the EP4 receptor and thus inhibiting downstream inflammatory responses (Kabashima et al., 2003; Jones et al., 2009; Esaki et al., 2010). Local inflammatory responses in the peritoneum contribute to deterioration of peritoneal transport function and peritoneal fibrosis (de Lima et al., 2013). The results of our *in vivo* experiments showed that ONO-AE3-208 reduced the high-glucose–induced inflammatory response in the peritoneum and peritoneal fibrosis, thereby reducing peritoneal pathologic injury and improving peritoneal function. Our *in vivo* experiments also revealed that ONO-AE3-208 had little effect on EP4 in the peritoneum. Although ONO-AE3-208 reduced the peritoneal inflammatory response, the peritoneum was still surrounded by PD fluid containing a high concentration of glucose; therefore, increased expression of EP4 was still observed in PD rats treated with ONO-AE3-208. The EP4 antagonist can be administered orally and is not associated with changes in blood pressure (Aringer et al., 2018); therefore, clinical application of the EP4 antagonist is more promising. To date, the novel EP4 receptor antagonist has been studied in phase II clinical trials in patients with rheumatoid arthritis (ClinicalTrials.gov Identifier: NCT03163966) and patients with advanced prostate, breast, or non-small cell lung cancers (ClinicalTrials.gov Identifier: NCT02538432). Although our current results for ONO-AE3-208 are promising, further research will be necessary to investigate the efficacy and safety of different doses of this antagonist for the prevention and treatment of PD-associated peritoneal fibrosis.

The results of our previous study showed that mPGES-1 promoted the synthesis of extracellular matrix protein *via* activation of the NLRP3 inflammasome in RPMCs exposed to high levels of glucose (Luo et al., 2021), indicating that the NLRP3 inflammasome participated in peritoneal fibrosis. EP4 is one of the main downstream receptors of mPGES-1. Previous studies have shown that the PGE2/EP4 pathway is closely related to the activation of the NLRP3 inflammasome. For example, the PGE2 synthetic pathway was shown to promote albumin-induced renal tubular injury *via* an NLRP3 inflammasome-mediated mechanism (Zhuang et al., 2017). *Tityus serrulatus* venom triggered lung resident cells to release PGE2, which induced activation of the NLRP3 inflammasome *via* EP4-cAMP-PKA-NF-κB-dependent mechanisms, leading to an increase in IL-1β (Zoccal et al., 2016). In the present study, we further demonstrated that an EP4 antagonist attenuated peritoneal fibrosis by inhibiting activation of the NLRP3 inflammasome. Moreover, EP4 signalling has been shown to regulate NF-κB signalling. EP4 stimulation increased IL-6 production *via* PKA-NF-κB pathways in vascular smooth muscle cells isolated from the mouse aorta and human abdominal aortic aneurysms (Hiromi et al., 2020). Peptidoglycan-mediated IL-6 production was mediated by the PGE2/EP4/NF-κB pathway in RAW 264.7 macrophages (Chen et al., 2006). We further demonstrated that EP4 signalling regulated NF-κB signalling in RPMCs exposed to high concentrations of glucose.

In summary, we confirmed that the EP4 receptor promotes peritoneal fibrosis and that pharmacologic blockade of EP4 protects against peritoneal injury. EP4 was highly enhanced in the fibrotic peritoneum of PD patients with UFF and in cultured RPMCs stimulated with high glucose concentrations. Treatment with ONO-AE3-208 significantly prevented peritoneal inflammation and peritoneal fibrosis by inhibiting activation of the NLRP3 inflammasome and reducing phosphorylation of NF-κB. Therefore, EP4 inhibition may be a new strategy for the prevention and treatment of PD-associated peritoneal fibrosis in the future.

## Data Availability

The original contributions presented in the study are included in the article/Supplementary Material, further inquiries can be directed to the corresponding authors.
