# Supplementary material for: Blockade of prostaglandin E2 receptor 4 ameliorates peritoneal dialysis-associated peritoneal fibrosis
Source: Front Pharmacol. 2022 Nov 11;13:1004619. doi: 10.3389/fphar.2022.1004619 (PMC9691893; doi:10.3389/fphar.2022.1004619)

SUPPLEMENTARY FIGURE 1. Effects of different glucose concentrations on cell viability and effects of mannitol on EP4 expression in RPMCs. (A) Cell viability from RPMCs treated with different doses of glucose was examined by CCK-8. (B) Cell lysates from RPMCs treated with 138 mmol/L mannitol at different times were subjected to western blotting analysis with specific antibodies against EP4 and β-actin. (C) Expression levels of EP4 were quantified by densitometry, normalised with β-actin, and presented as fold changes. Data are presented as mean ± SEM. *P<0.05.

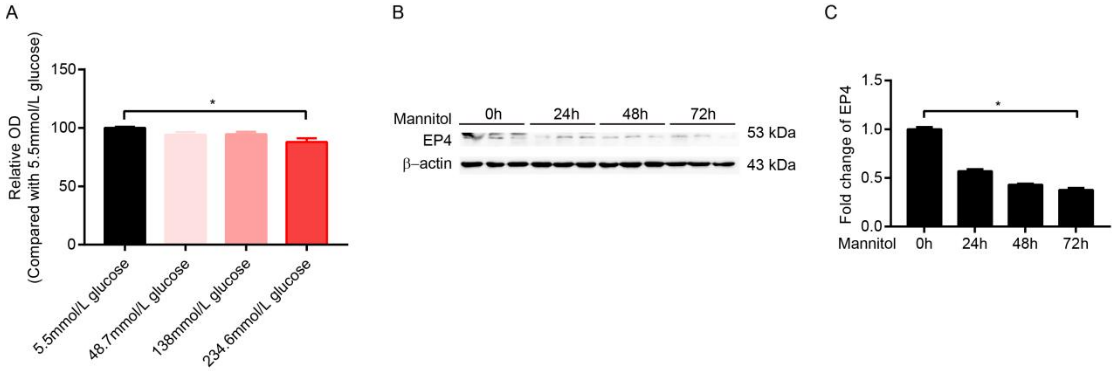

Supplement: Supplementary file 1 [file Image1.pdf]
